# Functional Analysis of the *Gibberellin 2-oxidase* Gene Family in Peach

**DOI:** 10.3389/fpls.2021.619158

**Published:** 2021-02-17

**Authors:** Jun Cheng, Jingjing Ma, Xianbo Zheng, Honglin Lv, Mengmeng Zhang, Bin Tan, Xia Ye, Wei Wang, Langlang Zhang, Zhiqian Li, Jidong Li, Jiancan Feng

**Affiliations:** College of Horticulture, Henan Agricultural University, Zhengzhou, China

**Keywords:** *Prunus persica*, peach, GA2ox, functional divergence, GA homeostasis

## Abstract

Peach (*Prunus persica* L. Batsch) trees grow vigorously and are subject to intense pruning during orchard cultivation. Reducing the levels of endogenous gibberellins (GAs) represents an effective method for controlling branch growth. Gibberellin 2-oxidases (GA2oxs) deactivate bioactive GAs, but little is known about the GA2ox gene family in peach. In this study, we identified seven *PpGA2ox* genes in the peach genome, which were clustered into three subgroups: C_19_-GA2ox-I, C_19_-GA2ox-II, and C_20_-GA2ox-I. Overexpressing representative genes from the three subgroups, *PpGA2ox-1*, *PpGA2ox-5*, and *PpGA2ox-2*, in tobacco resulted in dwarf plants with shorter stems and smaller leaves than the wild type. An analysis of the GA metabolic profiles of the transgenic plants showed that PpGA2ox-5 (a member of subgroup C_19_-GA2ox-II) is simultaneously active against both C_19_-GAs and C_20_-GAs,which implied that C_19_-GA2ox-II enzymes represent intermediates of C_19_-GA2oxs and C_20_-GA2oxs. Exogenous GA_3_ treatment of shoot tips activated the expression of all seven *PpGA2ox* genes, with different response times: the *C*_19_-*GA2ox* genes were transcriptionally activated more rapidly than the *C_20_-GA2ox* genes. GA metabolic profile analysis suggested that C_20_-GA2ox depletes GA levels more broadly than C_19_-GA2ox. These results suggest that the *PpGA2ox* gene family is responsible for fine-tuning endogenous GA levels in peach. Our findings provide a theoretical basis for appropriately controlling the vigorous growth of peach trees.

## Introduction

Peach (*Prunus persica* L.) is one of the most widely cultivated deciduous fruit trees worldwide. According to FAOSTAT data, the global cultivation area of peach and nectarine reached 1712.4 ha in 2018. Peach trees must be pruned to keep the tree size manageable and to facilitate harvesting, but most cultivated peach varieties grow vigorously, increasing the labor costs required for orchard management. One effective way to inhibit the rapid growth of peach branches is to treat the trees with paclobutrazol (PBZ), an inhibitor of gibberellin (GA) biosynthesis, indicating that endogenous GA is at least one of the phytohormones that promote the vigorous growth of peach branches.

The biosynthesis of GA has been studied in many plant species, and is catalyzed by seven enzymes: *ent*-copalyl diphosphate synthase (CPS), *ent*-kaurene synthase (KS), *ent*-kaurene oxidase (KO), *ent*-kaurenoic acid oxidase (KAO), GA 13-oxidase (GA13ox), GA 20-oxidase (GA20ox), and GA 3-oxidase (GA3ox) (Yamaguchi, 2008). Because GA homeostasis is crucial for proper plant growth and development, several mechanisms have evolved to reduce the levels of bioactive GAs in plants (Varbanova et al., 2007; Gao et al., 2016). The main mechanism involves catabolism through the 2β-hydroxylation of active GA, a process catalyzed by GA 2-oxidase (GA2ox) (Rieu et al., 2008).

GA2oxs belong to the 2OG-Fe (II) oxygenase superfamily (Rieu et al., 2008). GA2oxs are encoded by small gene families, as revealed in plants such as *Arabidopsis thaliana* (Schomburg et al., 2003; Rieu et al., 2008), rice (Lo et al., 2008), grapevine (Giacomelli et al., 2013), and tomato (Serrani et al., 2007). In Arabidopsis, *GA2ox* genes are expressed throughout plant development and in different tissues (Rieu et al., 2008). The role of GA2oxs in reducing the level of bioactive GAs has been demonstrated in many species, including rice (Lo et al., 2008), poplar (Gou et al., 2011), spinach (Lee and Zeevaart, 2005), and switchgrass (Wuddineh et al., 2015).

The expression levels of *GA2ox* genes vary in response to environmental changes and phytohormones. In Arabidopsis, bioactive GA is deactivated in the hypocotyls of light-grown seedlings via the activation of *GA2ox1* transcription (Achard et al., 2007). Blue light-mediated hypocotyl elongation is promoted by repressing the transcription of *GA2ox1* and *GA2ox8* (Wang et al., 2011). Low temperature deactivates GA by activating the transcription of *GA2ox* genes in rice (Wang et al., 2018). *GA2ox6* genes are upregulated under water-limiting conditions (Dubois et al., 2013). *GA2ox7* is upregulated by high salinity, which results in lower levels of active GAs (Magome et al., 2008). Finally, *GA2oxs* are significantly upregulated in response to treatment with exogenous GA_3_ (Thomas et al., 1999; Tan et al., 2018). Therefore, regulating the expression of *GA2ox* genes is a vital way to control the levels of endogenous GAs in response to environmental changes.

The biosynthesis of GA produces intermediates (GA_12_, GA_15_, GA_24_, GA_9_, GA_53_, GA_44_, GA_19_, and GA_20_) in addition to bioactive GAs (GA_1_ and GA_4_). GAs are classified into different groups based on their number of carbon (C) atoms, including C_20_-GAs (such as GA_12_, GA_15_, GA_24_, GA_53_, GA_44_, and GA_19_) and C_19_-GAs (such as GA_9_, GA_20_, GA_1_, and GA_4_). GA2ox proteins are classified as C_20_-GA2ox or C_19_-GA2ox based on their preference for C_20_-GA or C_19_-GA substrates, respectively. Phylogenetic analysis of GA2ox proteins further divided the C_19_-GA2ox group into two subgroups (Lee and Zeevaart, 2005; Lo et al., 2008; Wuddineh et al., 2015), suggesting that members of these two C_19_-GA2ox subgroups do not have exactly the same functions.

In the current study, we identified seven *PpGA2ox* genes in the peach genome. Phylogenetic analysis clustered these genes into three subgroups: C_19_-GA2ox-I, C_19_-GA2ox-II, and C_20_-GA2ox-I. The overexpression of representative *PpGA2ox* genes from the three subgroups in tobacco resulted in a typical dwarf phenotype, with shorter stems and smaller leaves than the wild type. *PpGA2ox5*, a member of the C_19_-GA2ox-II subgroup, encodes an enzyme with activity against C_20_-GAs and C_19_-GAs. Our findings suggest that C_19_-GA2ox-II enzymes might be intermediate forms that arose during the divergence of the C_20_-GA2ox and C_19_-GA2ox lineages. Exogenous GA_3_ treatment of peach branches activated the expression of all seven *PpGA2ox* genes but with different time courses. *PpGA2ox* subgroup C_19_-GA2ox-I members were the earliest to be upregulated by this treatment, while *PpGA2ox* genes in subgroups C_19_-GA2ox-II and C_20_-GA2ox-I were activated later.

## Results

### Identification of Seven *PpGA2ox* Genes in the Peach Genome

**FIGURE 1:**
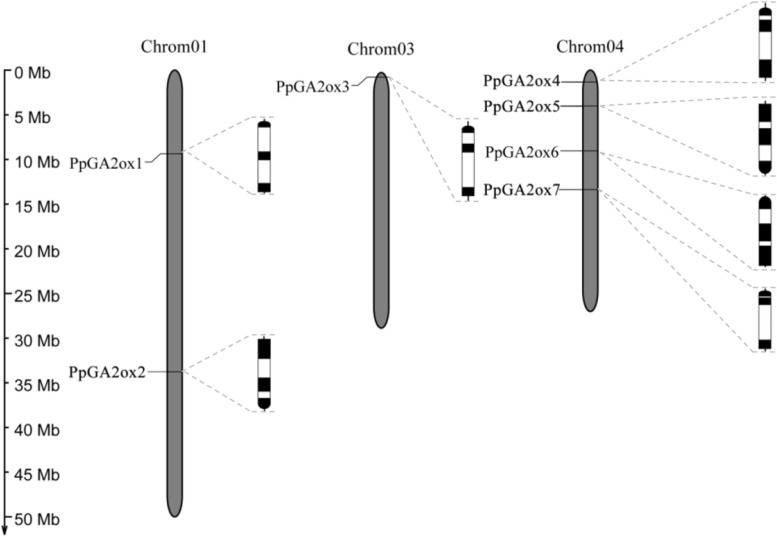
Chromosomal locations and genomic structures of *PpGA2ox* genes. Black boxes represent exons and white boxes represent introns. The semi-circle black boxes indicate the direction of the end of gene transcription.

We identified seven putative *GA2ox* genes in the peach genome: *PpGA2ox1*, *PpGA2ox2*, *PpGA2ox3*, *PpGA2ox4*, *PpGA2ox5*, *PpGA2ox6*, and *PpGA2ox7*. These genes are distributed on Chromosomes 1, 3, and 4 (Figure 1). All *PpGA2ox* genes contain three exons and two introns, which is similar to the structure of *GA2ox* family members in Arabidopsis (Han and Zhu, 2011). *PpGA2ox1* and *PpGA2ox2* on Chromosome 1 share 26% sequence identity and have opposite gene orientations. *PpGA2ox4*, *PpGA2ox5*, *PpGA2ox6*, and *PpGA2ox7* are located on Chromosome 4. The pairs *PpGA2ox5-PpGA2ox6* (65% identity) and *PpGA2ox4-PpGA2ox7* (52% identity) share the highest sequence identity among the *GA2ox* genes (Supplementary Table S1). *PpGA2ox4, PpGA2ox6*, and *PpGA2ox7* are oriented in the same direction, while *PpGA2ox5* is oriented in the opposite direction. These results suggest that intricate patterns of gene duplication occurred during the evolution of the peach *GA2ox* gene family.

Analysis of the conserved domains in the encoded enzymes revealed that the PpGA2oxs belong to the 2OG-Fe (II) oxygenase superfamily. All seven PpGA2oxs contain conserved amino acid residues that are thought to bind Fe^2+^ and 2-oxoglutarate (Supplementary Figure S1).

### Classification of the PpGA2oxs Into Three Subgroups: C_19_-GA2ox-I, C_19_-GA2ox-II, and C_20_-GA2ox-I

**FIGURE 2:**
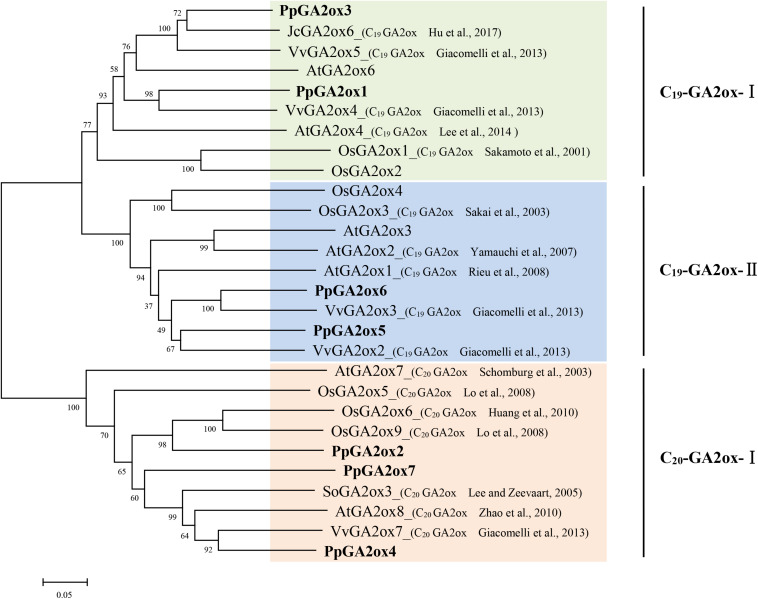
Phylogenetic tree of the predicted amino acid sequences of the GA2oxs from different species using MEGA5.1 program. The amino acid sequences formed three subgroup (C_19_-GA2ox-I, C_19_-GA2ox-II, and C_20_-GA2ox-I) and highlighted in different colors. Gene accession numbers of the sequences used in this tree are listed in Supplementary Table S2.

GA2oxs are usually active against C_19_-GAs (such as GA_9_, GA_20_, GA_1_, and GA_4_) or C_20_-GAs (such as GA_12_ and GA_53_), placing them into the C_19_-GA2ox or C_20_-GA2ox class (Rieu et al., 2008). To predict the functions of the PpGA2oxs, we collected reported GA2ox sequences from different plants and constructed a phylogenetic tree of the GA2ox family (Figure 2). The GA2ox family members fell into three groups: C_19_-GA2ox-I, C_19_-GA2ox-II, and C_20_-GA2ox-I. The reported members of C_19_-GA2ox-I and C_19_-GA2ox-II use C_19_-GAs as substrates, while the members in C_20_-GA2ox-I are mainly active against C_20_-GAs. PpGA2ox1 and PpGA2ox3 belong to subgroup C_19_-GA2ox-I; PpGA2ox5 and PpGA2ox6 belong to subgroup C_19_-GA2ox-II; and PpGA2ox2, PpGA2ox4, and PpGA2ox7 belong to subgroup C_20_-GA2ox-I. This clustering pattern predicts that PpGA2ox1, PpGA2ox3, PpGA2ox5, and PpGA2ox6 are active against C_19_-GAs, while PpGA2ox2, PpGA2ox4, and PpGA2ox7 are active against C_20_-GAs.

To determine whether the three subgroup division is conserved in other species, the reported GA2ox families from rice, grapevine, peach, tomato, and cucumber were selected and the number of *GA2ox* genes in each of the three subgroups was analyzed (Table 1). All species carry representatives of the C_19_-GA2ox-I, C_19_-GA2ox-II, and C_20_-GA2ox-I subgroups, suggesting that simultaneously possessing C_19_-GA2ox-I, C_19_-GA2ox-II, and C_20_-GA2ox-I genes is a highly conserved feature of both monocots and dicots.

**TABLE 1:** The number of *GA2ox* genes from six species distributed into the three subgroups.

	C19-GA2ox-I	C19-GA2ox-II	C20-GA2ox-I	References
Arabidopsis	2	3	2	Han and Zhu, 2011
Rice	2	4	4	
Cucumber	1	3	1	Pimenta Lange et al., 2013
Grapevine	2	3	4	Giacomelli et al., 2013
Tomato	2	5	4	Chen et al., 2016
Peach	2	2	3	

### Analysis of the Evolutionary Relationships Among C_19_-GA2ox-I, C_19_-GA2ox-II, and C_20_-GA2ox-I Genes

The functional divergence of genes is a common occurrence during evolution. We constructed a phylogenetic tree based on *GAox* genes from liverwort (*Marchantia polymorpha*), the bryophyte *Sphagnum fallax*, and the lycophyte *Selaginella moellendorffii* and *GA2ox* genes from the flowering plants rice (*Oryza sativa*), peach (*Prunus persica*), and *Arabidopsis thaliana* (Supplementary Figure S2). The first three species represent basal clades, with *Selaginella* representing the earliest vascular plants. All GAoxs from *M. polymorpha*, *Sph. fallax*, and *Sel. moellendorffii* clustered with the C_20_-GA2oxs from monocots and dicots. This result implies that C_20_-GA2ox is a basal clade of the GA2ox family that possesses more ancient gene functions.

**FIGURE 3:**
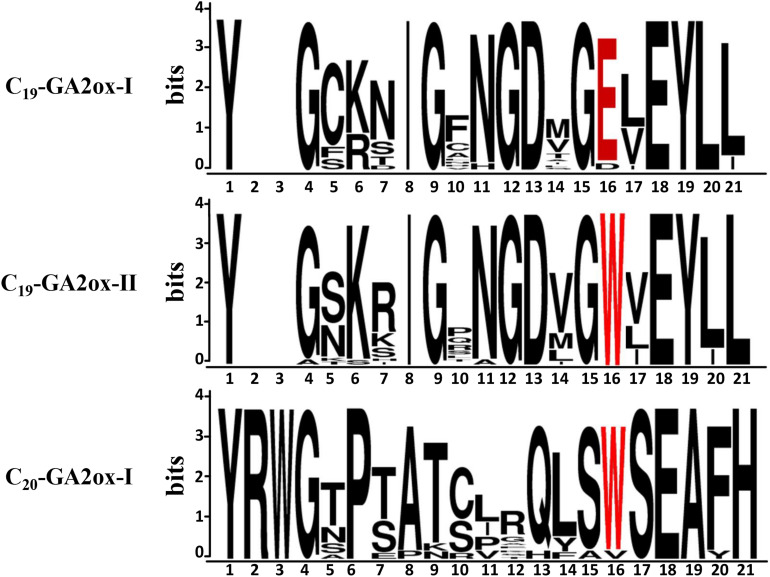
Graphical representation of the signature motif of GA 2-oxidases generated by WebLogo. Letter size is proportional to the degree of amino acid conservation. The 16th amino acid was highlighted in red font.

Motif analysis of GA2ox, GA20ox, and GA3ox proteins uncovered an amino acid sequence in the N-termini of these proteins that is the signature motif of GA2oxs (Han and Zhu, 2011), pointing to a close relationship between the signature motif and the functions of GA2oxs. To identify the possible cause of the functional divergence within the *GA2ox* family, we analyzed the signature motifs of the proteins in the C_19_-GA2ox-I, C_19_-GA2ox-II, and C_20_-GA2ox-I subgroups (Figure 3). The most highly conserved amino acids in C_19_-GA2ox-II proteins are the same as those in C_19_-GA2ox-I proteins, except for tryptophan (W; at position 16 in the wider multiple sequence alignment), which is highly conserved among C_20_-GA2ox-I proteins. This observation suggests that the C_19_-GA2ox-II subgroup is intermediate between the C_19_-GA2ox-I and C_20_-GA2ox-I subgroups.

### Transcriptional Profile of *PpGA2ox* Genes in Different Tissues

**FIGURE 4:**
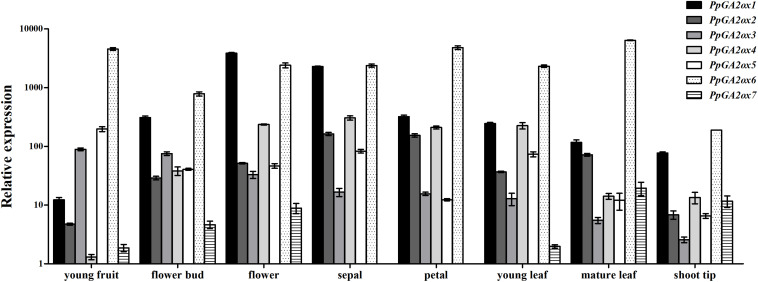
Expression of *PpGA2ox* genes in organs harvested from 3 year old “QMH” peach trees. Total RNA was isolated and analyzed by qRT-PCR, and the PpTEF2 was used as a reference gene. Results are plotted as the ratio to the lowest detected level (PpGA2ox7 in sepal). Note that the *y*-axis is on a logarithmic scale. All data are means of three replicates with error bars indicating standard deviation.

To investigate the expression profile of *PpGA2ox* genes in different tissues, we analyzed their transcript levels in six tissues (young fruit, flower bud, flower, young leaf, mature leaf, and shoot tip tissue) (Figure 4); *PpTEF2* was used for normalization. *PpGA2ox7* was expressed at the lowest level in all tissues analyzed compared to the six other *PpGA2ox* genes. *PpGA2ox1* was expressed at higher levels in flowers and sepals than in other tissues. *PpGA2ox2* and *PpGA2ox4* were expressed at higher levels in sepals compared to other tissues. *PpGA2ox1*, *PpGA2ox2*, and *PpGA2ox4* were expressed at relatively high levels in flower tissue. *PpGA2ox3* and *PpGA2ox5* were expressed at higher levels in young fruits compared to other tissues. *PpGA2ox6* was expressed at lower levels in shoot tips compared to other tissues.

### Overexpressing *PpGA2ox* Genes in Tobacco Causes a Dwarf Phenotype

**FIGURE 5:**
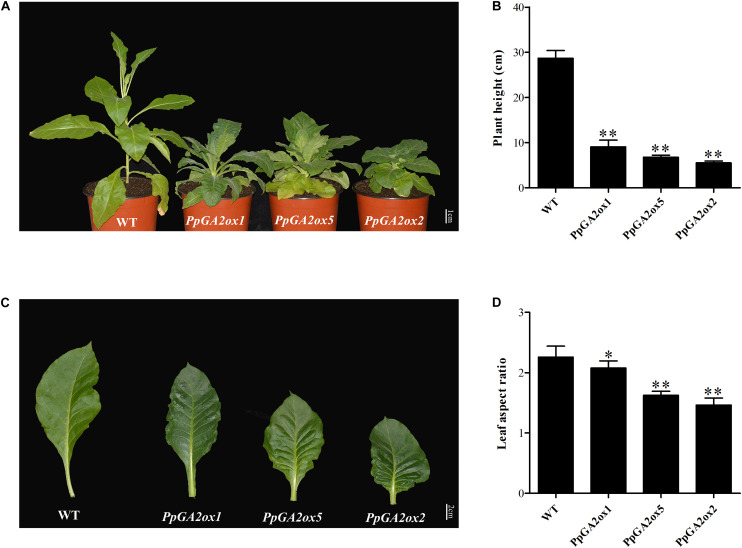
Ectopic expression of *PpGA2ox1, PpGA2ox5*, and *PpGA2ox2* in tobacco induces dwarfism and smaller leaves. **(A)** Transgenic tobacco plants were grown in a climate chamber for 3 months. **(B)** Heights of the WT and transgenic tobacco plants. **(C)** Mature leaves of WT and transgenic tobacco. **(D)** Leaf aspect ratios of the WT and transgenic tobacco plants. The values are presented as the means ± standard deviation. ^∗^Significantly different from the wild type at *P* < 0.05. ^∗∗^Significantly different from the wild type at *P* < 0.01.

To clarify the function of a representative peach *GA2ox* gene from each of the three subgroups (C_19_-GA2ox-I, C_19_-GA2ox-II, and C_20_-GA2ox-I), we generated the *35S:PpGA2ox1*, *35S:PpGA2ox5*, and *35S:PpGA2ox2* constructs and introduced them into tobacco by *Agrobacterium-*mediated transformation. For each construct, more than three independent transgenic tobacco lines were obtained, which were confirmed by PCR and qRT-PCR (Supplementary Figure S3). All transgenic plants exhibited dwarf phenotypes compared to the non-transgenic tobacco control (WT) (Figure 5A). The heights of the transgenic plants were no more than 30% that of the WT (Figure 5B). In addition, the mature leaves of the transgenic plants were crinkled and smaller than those of the WT (Figures 5C,D). These results suggest that PpGA2ox1, PpGA2ox5, and PpGA2ox2 negatively regulate plant height and strongly affect leaf development.

### PpGA2ox5 Is Active Against Both C_20_-GAs and C_19_-GAs

**FIGURE 6:**
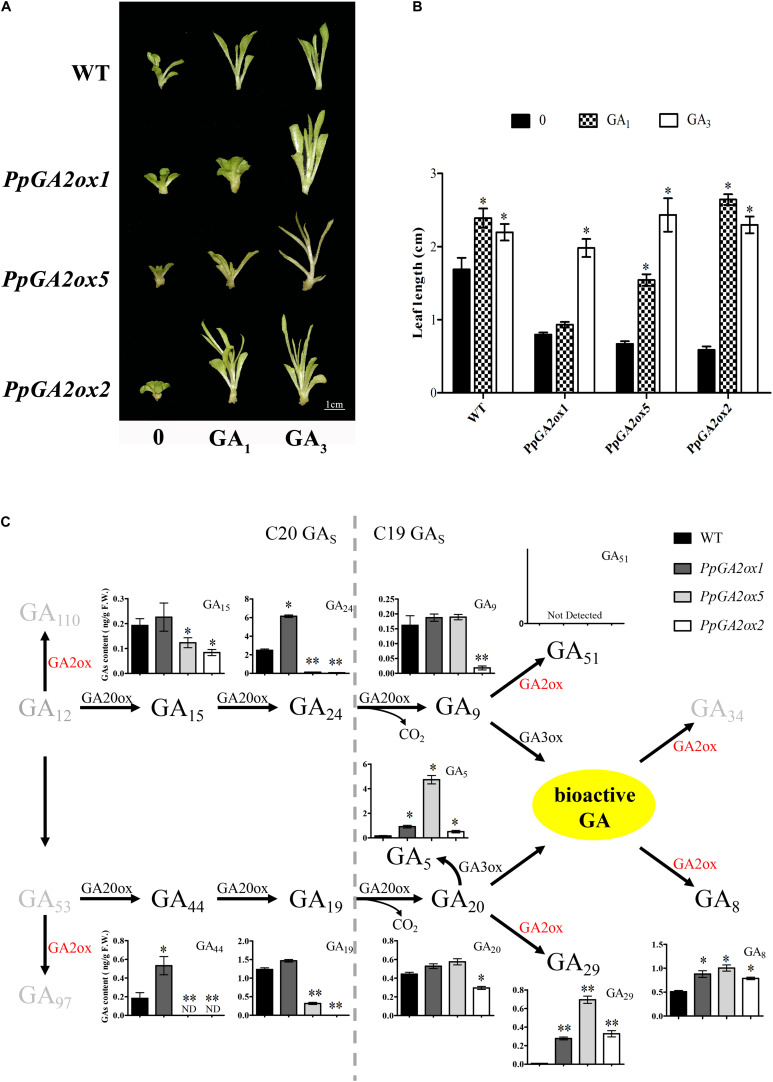
Analysis the function of PpGA2ox1, PpGA2ox5, and PpGA2ox2. **(A,B)** Effects of GA_1_ and GA_3_ on phenotypes of transgenic plants. Shoots cultivated in MS medium containing 0.4 or 0.0 mg/L GA_1_ or GA_3_. WT represents the non-transgenic plant. Bar represents 1 cm. **(C)** Effects of PpGA2ox1, PpGA2ox5, and PpGA2ox2 on the biosynthesis pathway of GA in tobacco measured using HPLC-MS/MS. The values are presented as the means ± standard error. ^∗^Significantly different from the wild type or untreated plant (*P* < 0.05). ^∗∗^Significantly different from the wild type (*P* < 0.01).

Based on our phylogenetic analysis (Figure 2), we predicted that PpGA2ox1 and PpGA2ox5 would be active against C_19_-GAs, while PpGA2ox2 would be active against C_20_-GAs. To elucidate the different metabolic functions of these enzymes, we analyzed the responses of the PpGA2ox1-OE, PpGA2ox5-OE, and PpGA2ox2-OE tobacco lines to exogenous GA_1_, a C_19_-GA (Figures 6A,B). GA_3_, which is resistant to enzyme-catalyzed degradation by GA2ox due to the presence of a double bond between C-2 and C-3 (Kanno et al., 2016), was used as a positive control. The wild type and all transgenic plants showed rapid growth after GA_3_ treatment. By contrast, after GA_1_ treatment, wild-type plants and plants overexpressing *PpGA2ox2* showed rapid growth, whereas plants overexpressing *PpGA2ox1* showed no significant difference in growth from the negative control (PpGA2ox1-OE with 0 mg/L GA_1_ treatment). These results strongly suggest that PpGA2ox1 is a C_19_-GA2ox and that PpGA2ox2 is a C_20_-GA2ox. Interestingly, plants overexpressing *PpGA2ox5* showed an intermediate phenotype compared to the PpGA2ox1-OE and PpGA2ox2-OE lines. These results suggest that PpGA2ox5 is active against GA_1_. However, PpGA2ox5 is less efficient than PpGA2ox1, suggesting that PpGA2ox5 might not be a classical type of C_19_-GA2ox.

To determine how PpGA2ox5 may differ from PpGA2ox1 and PpGA2ox2, we analyzed the GA metabolic profiles in shoot tips with young leaves from wild-type tobacco plants and transgenic lines overexpressing *PpGA2ox1*, *PpGA2ox2*, or *PpGA2ox5* (Figure 6C). We measured the contents of four C_20_-GAs (GA_15_, GA_24_, GA_44_, and GA_19_) and seven C_19_-GAs (GA_5_, GA_8_, GA_9_, GA_20_, GA_29_, GA_34_, and GA_51_) using HPLC-MS/MS. The levels of the four C_20_-GAs (GA_15_, GA_24_, GA_4_, and GA_19_) were higher in the PpGA2ox1-OE line and lower in the PpGA2ox5-OE and PpGA2ox2-OE lines vs. the wild type. GA_24_ and GA_44_ levels were significantly higher in the PpGA2ox1-OE line, whereas the levels of all four C_20_-GAs were significantly lower in the PpGA2ox5-OE and PpGA2ox2-OE lines compared to the wild type. Only PpGA2ox2-OE plants showed significantly lower levels of both C_19_-GAs (GA_9_ and GA_20_) compared to the wild type. GA_8_ and GA_29_ levels were significantly higher in all transgenic plants compared to the wild type. GA_5_ levels were significantly higher in all three transgenic lines vs. the wild type as well. Moreover, GA_5_ contents were 30.3-fold higher in PpGA2ox5-OE than in the wild type. GA_12_, GA_110_, GA_97_, GA_51_, and GA_34_ were not detected in any of the plants. These results suggest that PpGA2ox5 is active not only against C_19_-GAs, but also against C_20_-GAs.

Surprisingly, there were no differences in GA_8_ content among PpGA2ox1-OE, PpGA2ox5-OE, and PpGA2ox2-OE plants, but PpGA2ox1-OE plants showed higher activity against GA_1_ compared to PpGA2ox5-OE and PpGA2ox2-OE. The main bioactive GA in tobacco is GA_4_, whereas GA_1_ levels are low in this plant (Xiao et al., 2016). This paradoxical result could be due to the different concentrations of GA_1_ (endogenous vs. exogenous GA_1_) in the plants.

### C_19_-GA 2-Oxidases Are Transcriptionally Activated More Rapidly Than C_20_-GA 2-Oxidases After GA_3_ Treatment

**FIGURE 7:**
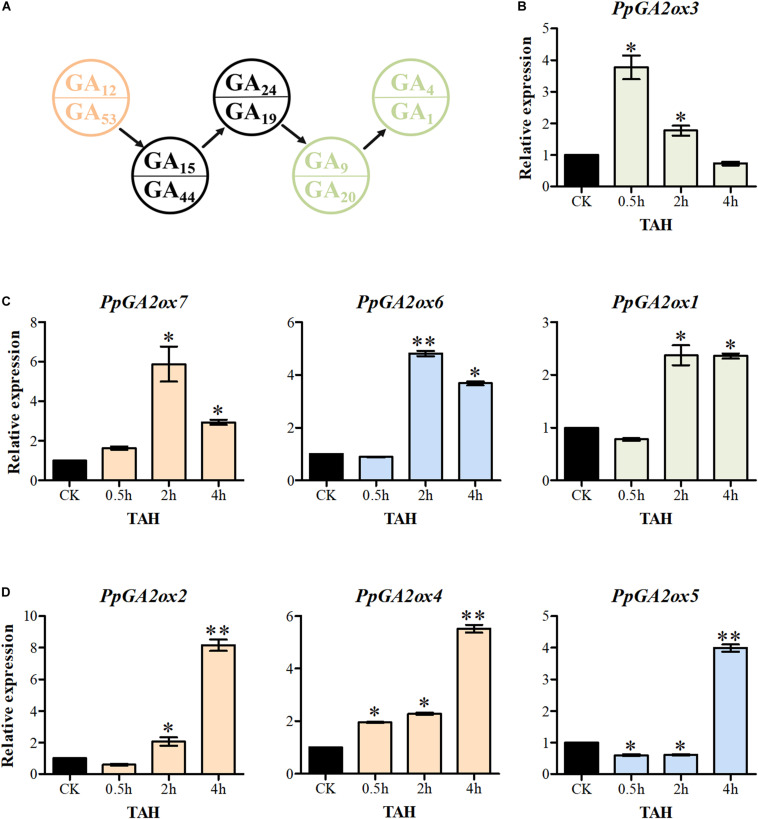
The transcription of *PpGA2ox* genes after GA_3_ treatment. **(A)** Part of GA biosynthesis way. Pink and green font represent the substrates for C_20_-GA2ox and C_19_-GA2ox, respectively. Seven *PpGA2ox* genes were divided into three type based on the response time to GA_3_ treatment. **(B)** The type 1 showed the most rapid response to GA_3_ treatment. **(C)** The type 2 reached to the peak transcript level at 2 h. **(D)** The type 3 reached to the peak transcript level at 4 h. Green, blue and pink columns represent the genes from the C_19_-GA2ox-I, C_19_-GA2ox-II, and C_20_-GA2ox-I subfamilies, respectively. TAH, treatment after hours. CK was the control treatment sprayed with distilled water. The values are presented as the means ± standard deviation. ^∗^Significantly different from the control (*P* < 0.05). ^∗∗^Significantly different from the control (*P* < 0.01).

Increased levels of bioactive GA significantly activate the transcription of *GA2oxs* in Arabidopsis (Rieu et al., 2008). In the current study, overexpressing a C_20_-GA2ox reduced the levels of GA_15_, GA_24_, GA_44_, GA_19_, GA_9_, and GA_20_ in tobacco, while overexpressing a C_19_-GA2ox promoted the accumulation of GA_24_ and GA_44_ (Figure 6C). These findings suggest that C_20_-GA2oxs and C_19_-GA2oxs play different roles in regulating the levels of bioactive GAs. We therefore investigated the responses of C_20_-GA2ox and C_19_-GA2ox genes in shoot tips to treatment with GA_3_ for 0.5, 2, and 4 h (Figure 7). The seven *PpGA2ox* genes were divided into three types based on their transcriptional responses. The type 1 response, which was observed only for *PpGA2ox3*, was the most rapid response to GA_3_ treatment, with peak expression at 0.5 h followed by a reduction at 2 and 4 h after GA_3_ treatment. The type 2 response, which was observed for *PpGA2ox1*, *PpGA2ox6*, and *PpGA2ox7*, was characterized by peak transcript levels at 2 h after GA_3_ treatment that remained high at 4 h after treatment. The type 3 response to GA_3_, as observed for *PpGA2ox2*, *PpGA2ox4*, and *PpGA2ox5*, was slower, with gradually increasing transcript levels at the first two time points and a peak at 4 h. These results suggest that the transcription of the C_19_-GA2ox genes is activated more rapidly than the transcription of C_20_-GA2ox genes after GA_3_ treatment.

## Discussion

GAs are important phytohormones that control diverse aspects of plant growth and development, including seed germination, stem elongation, leaf expansion, and flower and seed development (Yamaguchi, 2008). The homeostasis of bioactive GA levels is crucial for plant growth and development. GA2ox enzymes catalyze the deactivation of GA, thereby helping to maintain GA homeostasis. In the current study, we identified seven *PpGA2ox* genes in the peach genome. Our findings suggest that C_19_-GA2ox-II enzymes might be evolutionary intermediates between C_20_-GA2oxs and C_19_-GA2oxs and that the PpGA2ox family is responsible for maintaining the delicate balance of GA homeostasis.

### C_19_-GA2ox-II Enzymes Might Be Intermediates Between C_20_-GA2oxs and C_19_-GA2oxs

The GA2 oxidase family is divided into three groups: C_19_-GA2ox-I, C_19_-GA2ox-II, and C_20_-GA2ox-I. These groups are highly conserved in monocots and dicots (Table 1), suggesting there are likely functional differences among the groups. Here, we demonstrated that PpGA2ox5 belongs to subfamily C19-GA2ox-II and is simultaneously active against both C_20_-GAs and C_19_-GAs. Tobacco plants overexpressing PpGA2ox5 contained lower levels of four C_20_-GAs (GA_15_, GA_24_, GA_44_, and GA_19_) than the wild type, which was similar to the results for PpGA2ox2-OE (a predicted C_20_-GA2ox) but opposite to those for of PpGA2ox1-OE (a predicted C_19_-GA2ox). These changes in C_20_-GA levels in the PpGA2ox5-OE lines were consistent with those in Arabidopsis *c_19_-ga2ox* and *c_20_-ga2ox* mutants (Magome et al., 2008; Rieu et al., 2008). In addition, PpGA2ox5-OE contained significantly higher levels of GA_5_ and GA_29_ than the wild type. These results suggest that PpGA2ox5-OE is active against C_19_-GAs.

PpGA2ox1-OE showed higher levels of GA_15_, GA_24_, GA_44_, and GA_19_ than the WT. Perhaps these plants exhibited increased GA 20-oxidase activity resulting from feedback regulation due to reduced levels of bioactive GAs. PpGA2ox2-OE also contained significantly higher levels of GA_5_ and GA_29_ than the wild type, suggesting that PpGA2ox2 also has activity against C_19_-GAs. While PpGA2ox2-OE plants had a dwarf phenotype, they grew rapidly after GA_1_ treatment. These results suggest that PpGA2ox2-OE might also have two activities against C_20_-GAs and C_19_-GAs, although its major activity is against C_20_-GAs. VvGA2ox7, a member of C_20_-GA2ox-I in grapevine, is also active against C_19_-GAs (GA_9_, GA_20_, GA_4_, and GA_1_) (Giacomelli et al., 2013).

Understanding the evolutionary relationships among gene subfamilies can uncover information about the course of functional divergence of genes. C_19_-GA2oxs, C_20_-GA2oxs, and GA biosynthetic oxidases (GA20ox and GA3ox) are all members of the 2OG-Fe (II) oxygenase superfamily (Han and Zhu, 2011). Although the evolutionary relationships among the C_19_-GA2ox, C_20_-GA2ox, GA20ox, and GA3ox subfamilies is unclear, the four subfamilies likely share a common ancestor (Giacomelli et al., 2013; Huang et al., 2015). Our results suggest that C_20_-GA2ox-I is an older clade of the GA2ox family compared to C_19_-GA2ox. Indeed, several studies have suggested that C_19_-GA2ox genes were derived from C_20_-GA2ox genes (Han and Zhu, 2011; Giacomelli et al., 2013; Huang et al., 2015). In an addition, only *ent*-kaurenoic acid, a gibberellin precursor, was detected in *Physcomitrella patens*, and *ent*-kaurenoic acid oxidase (KAO), GA20ox, and GA3ox were absent in this moss (Hirano et al., 2007; Miyazaki et al., 2018). *ent*-kaurenoic acid contains 20 carbons and can be converted into the bioactive GAs by KAO, GA20ox, and GA3ox. This observation suggested that C_20_-GA arose before C_19_-GA during evolution, implying that enzymes active against C_20_-GAs evolved earlier than enzymes active against C_19_-GAs.

**FIGURE 8:**
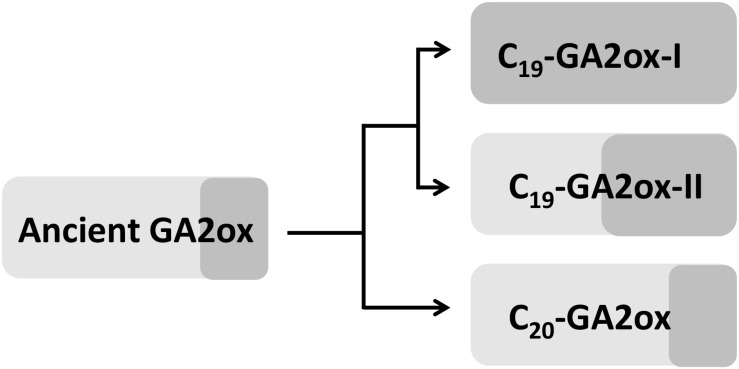
Hypothesis for the evolution of GA2 oxidases in higher plants. Light and dark gray box represents the activity of GA2ox against C_20_-GAs and C_19_-GAs, respectively. The area of box is proportional to the degree of enzyme activity.

Since PpGA2ox2 showed activity against C_19_-GAs, perhaps the ancient GA2ox was able to catalyze the 2β-hydroxylation of both C_19_-GAs and C_20_-GAs but was mainly active against C_20_-GAs and was only weakly active against C_19_-GAs. Perhaps once the ancient GA2ox evolved into two clades, one of the clades kept the original activity and formed the C_20_-GA2ox-I subfamily, while the other clade gradually gained increased activity against C_19_-GAs, resulting in the formation of the C_19_-GA2ox clade. The C_19_-GA2ox-II clade, containing PpGA2ox5, might have retained the ancient function of catalyzing the 2β-hydroxylation of C_20_-GAs (Figure 8).

Our analysis showed that the conserved tryptophan (W) in the signature motif of GA2ox is highly conserved in both C_19_-GA2ox-II and C_20_-GA2ox proteins, while a glutamic acid (E) is highly conserved in C_19_-GA2ox-I proteins. This tryptophan is also highly conserved in four clades of the 2OG-Fe (II) oxygenase superfamily (GA20ox, GA3ox, GAox-A, and GAox-C) (Huang et al., 2015). We speculate that this tryptophan is important for the ancient function of catalyzing the 2β-hydroxylation of the C_20_-GAs. More work is needed to verify this hypothesis.

### *PpGA2ox* Family Members Help Maintain the Delicate Balance of Endogenous GA Levels

Plants must cope with variable environmental conditions and are subjected to daily variations in light and temperature along with various biotic and abiotic stresses. Endogenous phytohormones help regulate plant growth and development and function in plant responses to the variable environment. GAs generally stimulate organ growth by enhancing cell elongation and cell division (Hedden and Thomas, 2012). The regulation of endogenous GA levels is important for regulating plant growth and development. The deactivation of GAs by GA2 oxidases is an important mechanism that influences GA responses. For example, bioactive GAs accumulate in the hypocotyls of dark-grown Arabidopsis seedlings and are deactivated in the hypocotyls of light-grown seedlings via the activated transcription of *GA2ox1* (Achard et al., 2007). Low temperature (15°C) promotes GA deactivation by activating the transcription of *GA2ox* genes in rice (Wang et al., 2018). High salinity upregulates *GA2ox7* in leaves, hypocotyls, and roots, resulting in reduced levels of active GAs (Magome et al., 2008). *GA2ox7* is also upregulated by cold stress (Zhou et al., 2017).

In the current study, we treated tobacco shoot tips with a high concentration of GA_3_, which appeared to be responsible for increasing the expression of *GA2ox* genes over time. The different response times of different *PpGA2ox* genes coupled with the different GA targets of the subgroups within the GA2ox family (C_19_-GA2ox-I, C_19_-GA2ox-II, and C_20_-GA2ox-I) illustrate how these enzymes fine-tune GA levels. Three *PpGA2ox* genes, one each from the C_19_-GA2ox-I, C_19_-GA2ox-II, and C_20_-GA2ox-I subgroups, were significantly upregulated after GA_3_ treatment at similar time points, whereas one gene from C_19_-GA2ox-II and one from C_20_-GA2ox-I were significantly upregulated more slowly after GA_3_ treatment. After GA_3_ treatment, the C_19_-GA2oxs were activated first, followed by the C_20_-GA2oxs. Six C_20_-GAs and four C_19_-GAs are intermediates in the GA biosynthesis pathway. The C_20_-GA2oxs significantly depleted the C_20_-GAs and some of the C_19_-GAs, while the C_19_-GA2oxs significantly depleted the C_19_-GAs but increased the levels of some of the C_20_-GAs (Figure 6). Ultimately, the C_20_-GA2oxs were activated, thereby blocking GA biosynthesis. These findings indicate that the plants employed an active response to cope with high GA_3_ treatment. Together, these results suggest that the *PpGA2ox* family contributes to the fine-tuning of endogenous GA levels via the different response times and different enzymatic activities of the individual PpGA2 oxidases.

## Materials and Methods

### Plant Materials and Growth Conditions

The peach cultivar used in this study, “QiuMiHong” (QMH), was provided by the Fruit Tree Germplasm Repository of Henan Agricultural University (Henan Province, China). For RNA extraction, young fruits, flower buds, flowers, sepals, petals, young leaves, mature leaves, and shoot tips were collected from 3 year old “QMH” trees in the spring, immediately frozen in liquid nitrogen, and stored at −80°C for subsequent experiments. Tobacco variety “K326” seedlings were grown *in vitro* in a temperature-controlled growth chamber at 24 ± 2°C under a 16 h light/8 h dark cycle. To calculate the leaf aspect ratio, a digital Vernier caliper was used to measure the distance from the bottom to the top of the leaf (length) and the maximum width of the leaf (width).

### Sequence Analysis and Cloning

To identify *GA2ox* genes in the peach genome, a BlastP with default parameters of the Phytozome database^1^ was performed based on the conserved domain of genes in the 2-oxoglutarate-dependent oxygenase family using the amino acid sequences of seven Arabidopsis GA2oxs as queries. The search results were aligned by ClustalW algorithm with default parameters to reduce duplicate and redundant sequences (Larkin et al., 2007). Gene information from the JGI database was then used to predict the genomic positions of the *PpGA2ox* genes. The *PpGA2ox* genes were renamed, plotted on the peach chromosomes, and visualized with MapGene2 Chrom^2^ (Jiangtao et al., 2015).

The amino acid sequences of all GA oxidases used in this study were downloaded from the Phytozome and NCBI databases^3^. Sequence logos were generated using the online WebLogo platform^4^ (Crooks et al., 2004). A phylogenetic tree was constructed using the neighbor-joining method (NJ) method in MEGA (version 5.1) (Tamura et al., 2011). The main parameters were set as follows: the model was set to “p-distance,” the GAPs was set to “pairwise deletion,” and bootstrapping was performed with 1000 replicates. Default parameters were used for all bioinformatics analyses, except for those specifically mentioned.

The full-length sequences of the *PpGA2ox* genes were cloned from RNA obtained from peach shoot tips using specific primers and ligated into the pEASY^®^-Blunt Vector (TransGen Biotech). PCR products that produced a single band on the gel were purified and sent to Sangon Biotech (Shanghai^5^) for sequencing. All primers used in this study are listed in Supplementary Table S3.

### Construction of the Overexpression Vector and Transformation of Tobacco

The full-length sequences of the *PpGA2oxs* were amplified from the pEASY^®^ -Blunt Vector (TransGen Biotech) using specific primers containing restriction sites and inserted into the pSAK277 vector containing the *CaMV35S* promoter. The plant overexpression vectors *35S:PpGA2ox1, 35S:PpGA2ox5*, and *35S:PpGA2ox2* were introduced into *Agrobacterium tumefaciens* strain GV3101 and used to transform tobacco leaf discs as described previously (Horsch et al., 1985; Lee and Zeevaart, 2005). Briefly, the leaves of aseptic tobacco seedlings were cut into small pieces (∼1 cm × 1 cm), pre-cultured in Murashige and Skoog (MS) medium for 2 days, incubated with *A. tumefaciens* (OD_600_ = 0.6) harboring the chosen construct for 5 min, and transferred to MS medium. After 2 days of co-culture in the dark, the leaf discs were transferred to selection medium (MS, 1 mg/L 6-benzylaminopurine, 0.1 mg/L NAA, 50 mg/L kanamycin, and 400 mg/L cefotaxime). When shoots appeared, individual shoots were cut and transferred to root-inducing medium (MS, 0.1 mg/L NAA, 50 mg/L kanamycin, and 400 mg/L cefotaxime). After rooting, the plantlets were grown in pots in a greenhouse and used for phenotypic analysis and further experiments. A pSAK277-GFP vector was transformed into tobacco in our lab and the transgenic lines showed no dwarf phenotype and smaller leaves.

### RNA Extraction and qRT-PCR Analysis

Total RNA was extracted from the samples using a Column-type Plant Total RNA Extraction and Purification Kit (Sangon Biotech. Shanghai, China). Purified total RNA (0.5–2 μg) was reverse transcribed into first-strand cDNA using a PrimeScript RT Reagent Kit with gDNA Eraser (TaKaRa Biotechnology, Beijing, China). The cDNA product was used for semiquantitative RT-PCR and qRT-PCR analysis. qRT-PCR analysis was conducted using SYBR Select Master Mix (Applied Biosystems, United States) according to the manufacturer’s protocol on an ABI PRISM 7500 FAST Sequence Detection System (Applied Biosystems, Madrid, CA, United States). All experiments were conducted in triplicate. To quantify relative transcript level, *TEF2* (Peach EST database accession number: TC3544) and β*-tubulin* (accession number: U91564) were used for data normalization (Tong et al., 2009; Schmidt and Delaney, 2010).

### Exogenous GA_3_ Treatment

One year old branches of “QMH” peach trees were sprayed with GA_3_ (100 mg/L) or with distilled water (as a control). Three biological replicates were performed for all treatments. Shoot tips were collected at 0.5, 2, and 4 h after treatment for RNA extraction to analyze *GA2ox* transcript levels.

### Quantification of Endogenous GAs

To measure endogenous phytohormone contents, approximately 1.0 g of young leaf tissue was collected from transgenic and wild-type tobacco plants and frozen in liquid nitrogen. The concentrations of different GAs were measured by high-performance liquid chromatography (HPLC) with tandem mass spectrometry (MS/MS) as described previously (Cheng et al., 2019). Three biological replicates were used to measure the level of each hormone.

### Treating Tobacco With GA_1_ and GA_3_

The shoots of transgenic tobacco were cultivated in MS medium containing 0.4 mg/L GA_1_ or 0.4 mg/L GA_3_. Changes in growth were observed 10 days after treatment. The lengths of 8–10 leaves were measured and averaged. The experiments were performed three times.

## Data Availability Statement

The datasets presented in this study can be found in online repositories. The names of the repository/repositories and accession number(s) can be found in the article/Supplementary Material.

## Author Contributions

JF, XZ, and JC conceived and designed the experiments. JM, HL, and MZ performed the experiments. JF and JC wrote the manuscript. BT, XY, and WW performed the GA metabolic analysis. LZ, ZL, and JL revised the manuscript. All authors contributed to the article and approved the submitted version.

## Conflict of Interest

The authors declare that the research was conducted in the absence of any commercial or financial relationships that could be construed as a potential conflict of interest.

## Footnotes

1 https://phytozome.jgi.doe.gov/pz/portal.html

2 http://mg2c.iask.in/mg2c_v2.0/

3 https://www.ncbi.nlm.nih.gov/

4 http://weblogo.berkeley.edu/logo.cgi

5 https://www.sangon.com/

## Supplementary Material

Supplementary Figure 1 Amino acid residues presumed to bind Fe at the active site (^∗^). Putative 2-oxoglutarate binding sites (▲) and the signature motif of GA2ox (Black overline). Two conserved domains DIOX_N (red overline) and 2OG-FeII_Oxy (green overline) were detected in all GA2ox proteins. Black shading indicates identity, magenta and cyan shading indicate that similar residues are greater than 75 and 50%, respectively.

Supplementary Figure 2 Analysis the evolutionary relationship of C_19_-GA2ox-I, C_19_-GA2ox-II and C_20_-GA2ox-I subgroups. A phylogenetic tree of GA2ox and GAox. All GA2oxs were searched through the Phytozome database (https://phytozome.jgi.doe.gov). Gene accession numbers of the sequences used in this tree are listed in Supplementary Table S2. ▲ represents the GA oxidases from liverwort (*Marchantia polymorpha*), *Sphagnum fallax*, *Selaginella moellendorffii*; ● represents the GA2 oxidases from rice, peach and *Arabidopsis thaliana.*

Supplementary Figure 3 qRT-PCR analysis of PpGA2ox-overexpressing transgenic and non-transgenic (WT) lines using RNA isolated from the tobacco young leaves. The expression levels of each gene were relative to Nt β-Tubulin. Bars represent mean values of three biological repeats indicated standard deviation. ^∗∗∗^Significantly different from the wild type (*P* < 0.001).

Supplementary Table 1 Comparison of the deduced amino acid sequences between PpGA2ox proteins.

Supplementary Table 2 The accession numbers of GAoxs used in this study.

Supplementary Table 3 Primer used in this study.
