# Supplementary figures and images for: Functional Analysis of the Gibberellin 2-oxidase Gene Family in Peach
Source: Front Plant Sci. 2021 Feb 17;12:619158. doi: 10.3389/fpls.2021.619158 (PMC7928363; doi:10.3389/fpls.2021.619158)

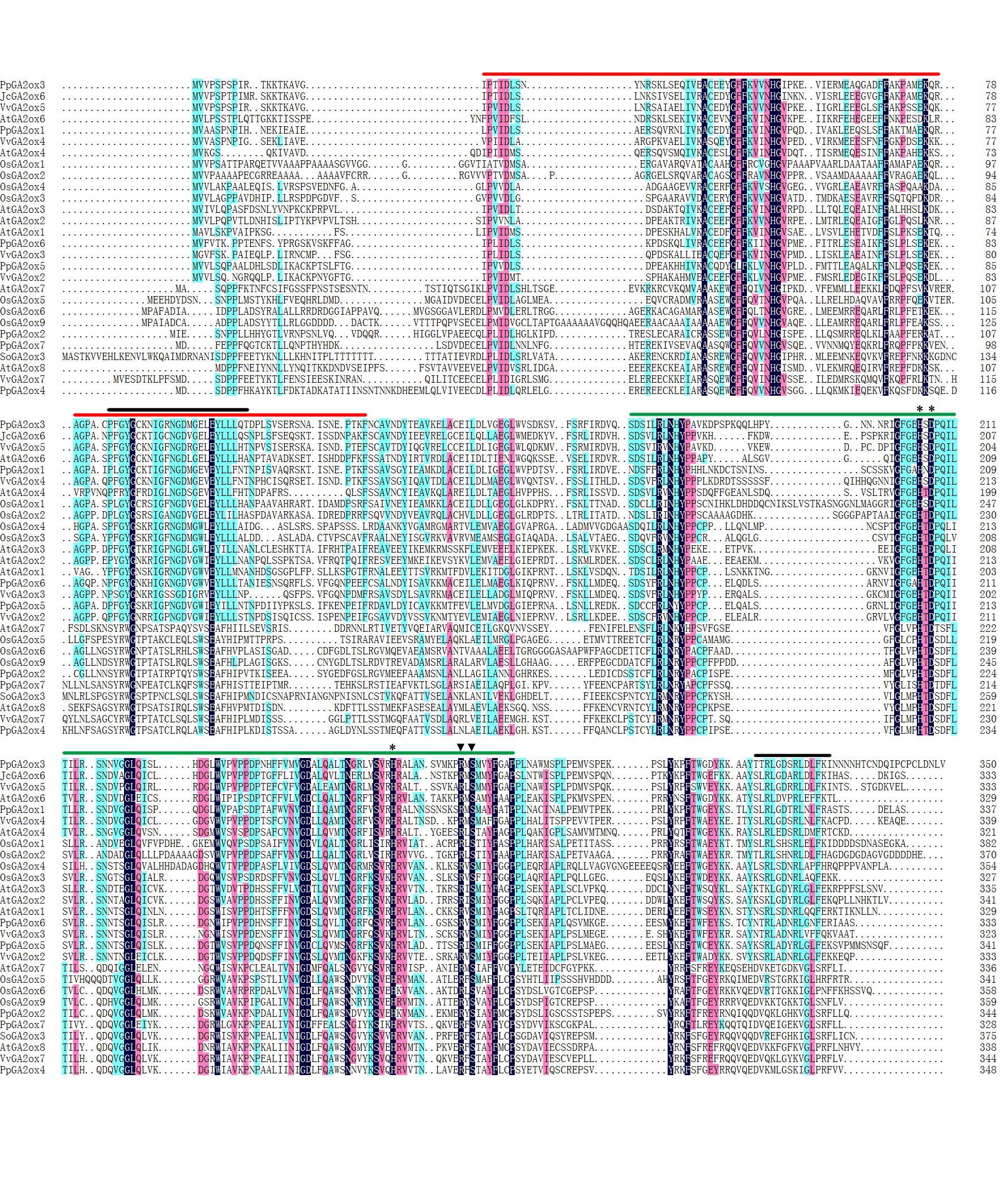

Supplement: Supplementary Figure 1 — Amino acid residues presumed to bind Fe at the active site (∗). Putative 2-oxoglutarate binding sites (▲) and the signature motif of GA2ox (Black overline). Two conserved domains DIOX_N (red overline) and 2OG-FeII_Oxy (green overline) were detected in all GA2ox proteins. Black shading indicates identity, magenta and cyan shading indicate that similar residues are greater than 75 and 50%, respectively. [file Image_1.TIF]

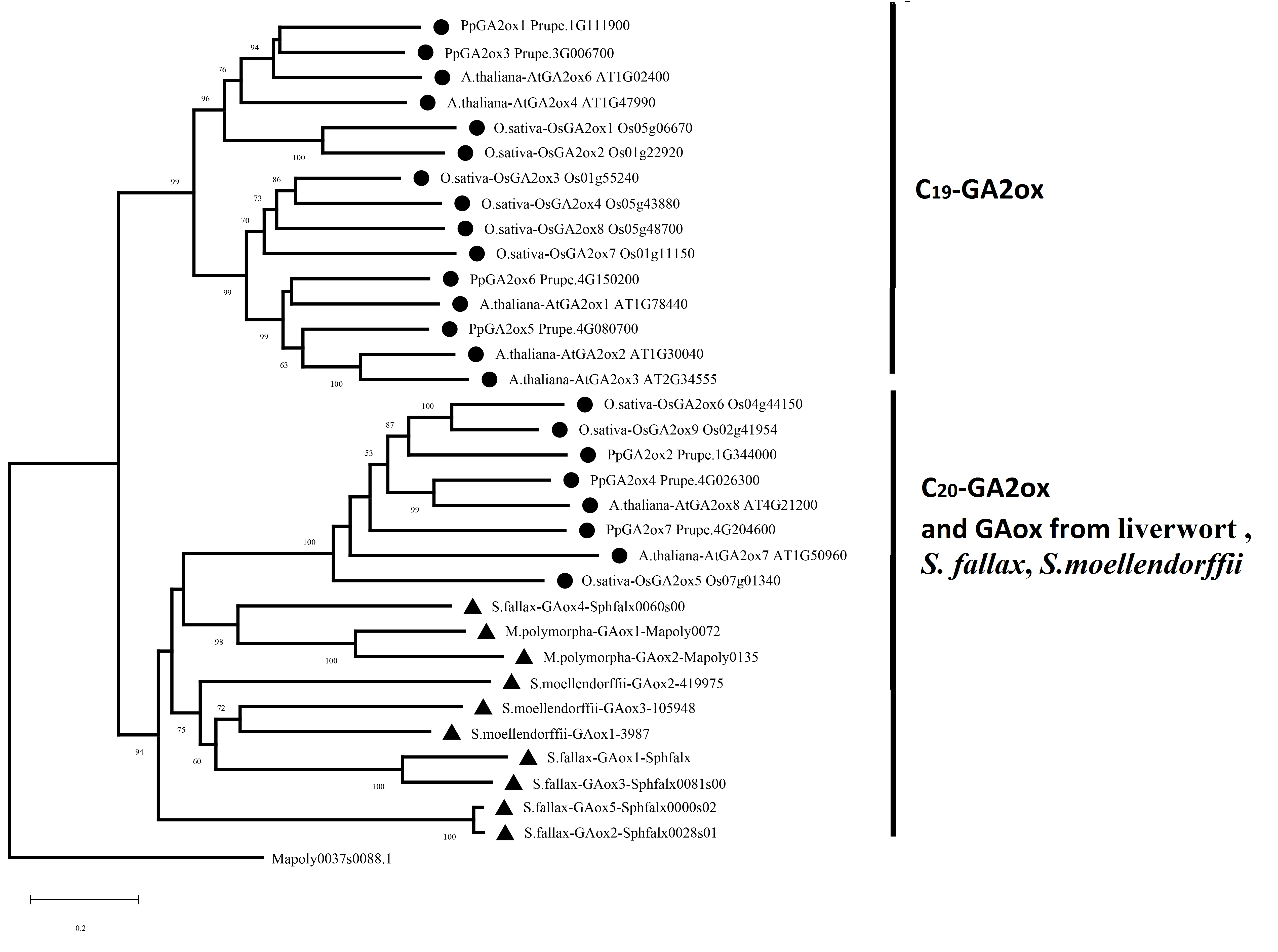

Supplement: Supplementary Figure 2 — Analysis the evolutionary relationship of C19-GA2ox-I, C19-GA2ox-II and C20-GA2ox-I subgroups. A phylogenetic tree of GA2ox and GAox. All GA2oxs were searched through the Phytozome database (https://phytozome.jgi.doe.gov). Gene accession numbers of the sequences used in this tree are listed in Supplementary Table S2. ▲ represents the GA oxidases from liverwort (Marchantia polymorpha), Sphagnum fallax, Selaginella moellendorffii; ● represents the GA2 oxidases from rice, peach and Arabidopsis thaliana. [file Image_2.TIF]

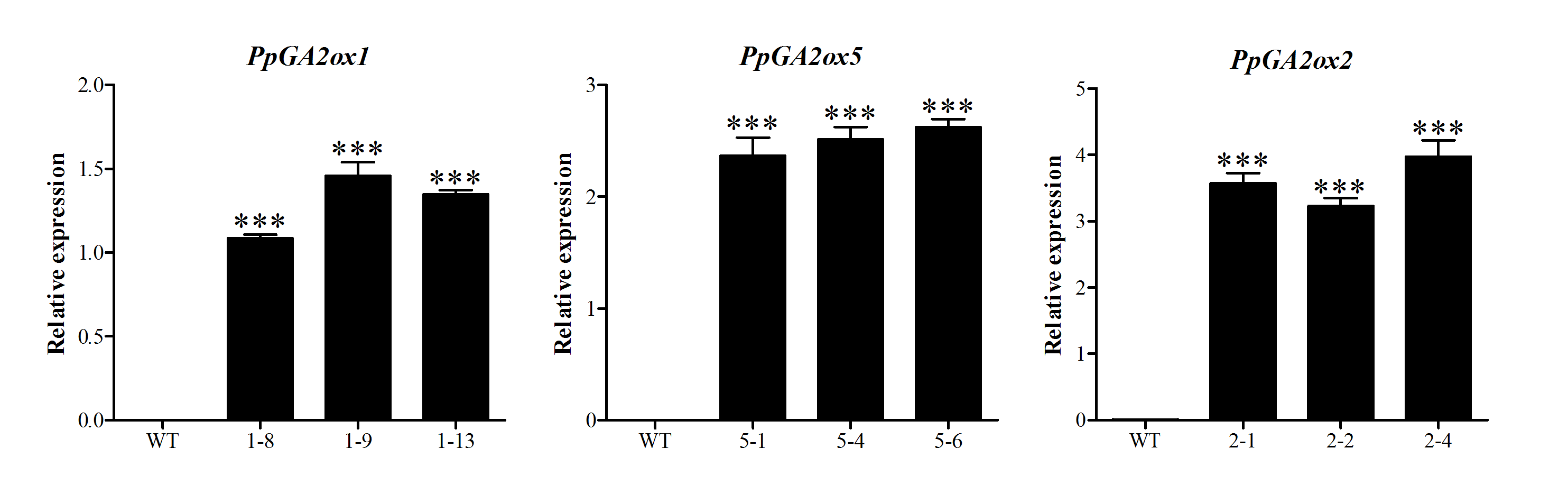

Supplement: Supplementary Figure 3 — qRT-PCR analysis of PpGA2ox-overexpressing transgenic and non-transgenic (WT) lines using RNA isolated from the tobacco young leaves. The expression levels of each gene were relative to Nt β-Tubulin. Bars represent mean values of three biological repeats indicated standard deviation. ∗∗∗Significantly different from the wild type (P < 0.001). [file Image_3.TIF]
