# Supplementary material for: Functional Analysis of the Gibberellin 2-oxidase Gene Family in Peach
Source: Front Plant Sci. 2021 Feb 17;12:619158. doi: 10.3389/fpls.2021.619158 (PMC7928363; doi:10.3389/fpls.2021.619158)
Supplement: Supplementary Table 1 — Comparison of the deduced amino acid sequences between PpGA2ox proteins. [file Data_Sheet_1.doc]

**Table 1 The number of GA2ox genes from six species distributed into the three subgroups.**

|  | C19-GA2ox-I | C19-GA2ox-II | C20-GA2ox-I | Reference |
| --- | --- | --- | --- | --- |
| Arabidopsis | 2 | 3 | 2 | Han and Zhu, 2011 |
| Rice | 2 | 4 | 4 |
| Cucumber | 1 | 3 | 1 | Chmielewska et al., 2013 |
| Grapevine | 2 | 3 | 4 | Giacomelli et al., 2013 |
| Tomato | 2 | 5 | 4 | Chen et al., 2016 |
| Peach | 2 | 2 | 3 |  |

**Table S1** Comparison of the deduced amino acid sequences between PpGA2ox proteins.

|  | PpGA2ox1 | PpGA2ox2 | PpGA2ox3 | PpGA2ox4 | PpGA2ox5 | PpGA2ox6 | PpGA2ox7 |
| --- | --- | --- | --- | --- | --- | --- | --- |
| PpGA2ox1 |  | 42% | 76% | 42% | 64% | 69% | 44% |
| PpGA2ox2 | 26% |  | 46% | 64% | 46% | 47% | 66% |
| PpGA2ox3 | 61% | 28% |  | 44% | 69% | 67% | 46% |
| PpGA2ox4 | 25% | 52% | 27% |  | 43% | 45% | 66% |
| PpGA2ox5 | 47% | 26% | 53% | 27% |  | 81% | 43% |
| PpGA2ox6 | 51% | 27% | 49% | 28% | 65% |  | 47% |
| PpGA2ox7 | 28% | 49% | 28% | 52% | 25% | 27% |  |

The numbers from upper right side and down left side indicate the percentage of positives and identities among predicted proteins.

**Table S****2** The accession numbers of GAoxs used in this study.

| **Database** | **Name** | **Species** | **Accession Number** | **Amino acid** |
| --- | --- | --- | --- | --- |
| Phytozome | AtGA2ox1 | *Arabidopsis thaliana* | AT1G78440 | 329 |
|  | AtGA2ox2 | *Arabidopsis thaliana* | AT1G30040 | 341 |
|  | AtGA2ox3 | *Arabidopsis thaliana* | AT2G34555 | 335 |
|  | AtGA2ox4 | *Arabidopsis thaliana* | AT1G47990 | 321 |
|  | AtGA2ox6 | *Arabidopsis thaliana* | AT1G02400 | 329 |
|  | AtGA2ox7 | *Arabidopsis thaliana* | AT1G50960 | 336 |
|  | AtGA2ox8 | *Arabidopsis thaliana* | AT4G21200 | 338 |
|  | OsGA2ox1 | *Oryza sativa* | LOC_Os05g06670 | 382 |
|  | OsGA2ox2 | *Oryza sativa* | LOC_Os01g22920 | 370 |
|  | OsGA2ox3 | *Oryza sativa* | LOC_Os01g55240 | 327 |
|  | OsGA2ox4 | *Oryza sativa* | LOC_Os05g43880 | 354 |
|  | OsGA2ox5 | *Oryza sativa* | LOC_Os07g01340 | 341 |
|  | OsGA2ox6 | *Oryza sativa* | LOC_Os04g44150 | 358 |
|  | OsGA2ox9 | *Oryza sativa* | LOC_Os02g41954 | 359 |
|  | VvGA2ox2 | *Vitis vinifera* | GSVIVT01021468001 | 333 |
|  | VvGA2ox3 | *Vitis vinifera* | GSVIVT01000687001 | 323 |
|  | VvGA2ox4 | *Vitis vinifera* | GSVIVT01034945001 | 339 |
|  | VvGA2ox5 | *Vitis vinifera* | GSVIVT01028169001 | 333 |
|  | VvGA2ox7 | *Vitis vinifera* | GSVIVT01012628001 | 344 |
|  | PpGA2ox1 | *Prunus persica* | Prupe.1G111900 | 337 |
|  | PpGA2ox2 | *Prunus persica* | Prupe.1G344000 | 344 |
|  | PpGA2ox3 | *Prunus persica* | Prupe.3G006700 | 351 |
|  | PpGA2ox4 | *Prunus persica* | Prupe.4G026300 | 348 |
|  | PpGA2ox5 | *Prunus persica* | Prupe.4G080700 | 341 |
|  | PpGA2ox6 | *Prunus persica* | Prupe.4G150200 | 333 |
|  | PpGA2ox7 | *Prunus persica* | Prupe.4G204600 | 328 |
|  | GAox1 | *Selaginellamoellendorffii* | 3987 | 276 |
|  | GAox2 | *Selaginellamoellendorffii* | 419975 | 360 |
|  | GAox3 | *Selaginellamoellendorffii* | 105948 | 397 |
|  | GAox1 | *Sphagnum fallax* | Sphfalx0218s0003 | 425 |
|  | GAox2 | *Sphagnum fallax* | Sphfalx0028s0151 | 401 |
|  | GAox3 | *Sphagnum fallax* | Sphfalx0081s0068 | 415 |
|  | GAox4 | *Sphagnum fallax* | Sphfalx0060s0044 | 397 |
|  | GAox5 | *Sphagnum fallax* | Sphfalx0000s0216 | 438 |
|  | GAox1 | *Marchantia polymorpha* | Mapoly0072s0026 | 392 |
|  | GAox2 | *Marchantia polymorpha* | Mapoly0135s0014 | 390 |
|  | Mapoly0037s0088.1 | *Marchantia polymorpha* | Mapoly0037s0088 | 372 |
| NCBI | SoGA2ox3 | *Spinacia oleracea* | AAX14674 | 375 |
|  | JcGA2ox6 | *Jatropha curcas* | KDP28294 | 333 |

**Table S3** Primer used in this study.

| **Primer name** | **Primer sequence 5′-3′** | **Purpose** |
| --- | --- | --- |
| GA2ox1F | CCCCATCATCTTTCTCTTCTTAGTT | Cloning |
| GA2ox1R | TTTTACTTTCCATTGCTGCCC |  |
| GA2ox2F | CCACCAAAGGACGACGAAG |  |
| GA2ox2R | AGGAAGGCAAGGTAAAGGAAAG |  |
| GA2ox3F | ATCACCTTGTTTTTCCCTTCATAA |  |
| GA2ox3R | CCATCAACCTTCAATTTCAACTACC |  |
| GA2ox4F | CCAAGTGTTATTTTCCAAGGTTTC |  |
| GA2ox4R | CCCTAATGATGTACTAGCAAAACTC |  |
| GA2ox5F | CTCTCAACATTCTCACCAAACCA |  |
| GA2ox5R | GCCTTCTATGGCAAACTGAGC |  |
| GA2ox6F | ACACGGAGGAGCAAGAACAACT |  |
| GA2ox6R | GGGAAGGGGGAAACAGAAGAG |  |
| GA2ox7F | TTTCCAGTAAACCCTATTTCTTCAT |  |
| GA2ox7R | CTGTTTTTCTTTAGGTCGTTTCATT |  |
| GA2ox1F- Kpn I | CGGGGTACCCCCCATCATCTTTCTCTTCTTAGTT | Overexpression |
| GA2ox1R- Xho I | CTCGAGTTTTACTTTCCATTGCTGCCC |  |
| GA2ox2F- Kpn I | CGGGGTACCCCACCAAAGGACGACGAAG |  |
| GA2ox2R- Xba I | TGCTCTAGAAGGAAGGCAAGGTAAAGGAAAG |  |
| GA2ox5F- Kpn I | CGGGGTACCCTCTCAACATTCTCACCAAACCA |  |
| GA2ox5R- Xho I | CCGCTCGAGGCCTTCTATGGCAAACTGAGC |  |
| GA2ox1cF | TTTGATTGGTGAGGGATTGTG | qRT-PCR |
| GA2ox1cR | GCTACTGTTGATGTTGCTGGTG |  |
| GA2ox2cF | GATTCCAAATGGGTTGCTGTGA |  |
| GA2ox2cR | AAGTAGGCGATTGAGTAGCGTTC |  |
| GA2ox3cF | TCAAGGACCCATCACCAAAAC |  |
| GA2ox3cR | AGGGACGGGAACCCACAA |  |
| GA2ox4cF | ATCAAGACCAGGTTGGAGGACT |  |
| GA2ox4cR | CTCATACGAAGGGCATAGGAAAT |  |
| GA2ox5cF | GCATAGGGTTTTGGCAGACAC |  |
| GA2ox5cR | GGACTTTTTGTATTCACACCACG |  |
| GA2ox6cF | CTTGAACTGATGGCTGAAGGATT |  |
| GA2ox6cF | GATGATTTGTGGGTCTGTGTGC |  |
| GA2ox7cF | GCCTACTTCTACTGTCCTTCCTACG |  |
| GA2ox7cR | AGTTCCTCATCTTTGGGCTTTGT |  |
| PpTEF2-F | GGTGTGACGATGAAGAGTGATG | Reference gene |
| PpTEF2-R | TGAAGGAGAGGGAAGGTGAAAG |  |
| Ntβ-Tubulin-F | GCATCTTTGCGTACACTTTGCT |  |
| Ntβ-Tubulin-R | ACATAAGCCCAAAACTAGCTGGA |  |
